# Supplementary material for: Patterns of Intron Gain and Loss in Fungi
Source: PLoS Biol. 2004 Nov 30;2(12):e422. doi: 10.1371/journal.pbio.0020422 (PMC532390; doi:10.1371/journal.pbio.0020422)
Supplement: Table S1 — Also available at http://genes.mit.edu/NielsenEtAl/. (4.3 MB ZIP). [file pbio.0020422.st001.zip › NielsenEtAl/html/485.html]

AN2248.1.NCU08998.1.MG01662.1.FG05554.1


```
 CLUSTAL W (1.82) Multiple Sequence Alignments - Introns Inserted


Sequence 1: NCU08998.1	515 aa
Sequence 2: MG01662.1	503 aa
Sequence 3: AN2248.1	498 aa
Sequence 4: FG05554.1	503 aa
Alignment Length: 518 aa
Number Identitical Residues: 279 aa
Alignment Score (without introns) 13362


MG01662.1 	MASVARGSALLRTLARPCSCSLARPALVPRALTRLAH-TKPFYS-------------EPE
NCU08998.1	MASRLLRSSYSGSLRAVSTASTVRPAAVS-AVHQFSN-RRQFTASSSKMASSFYKDWEPE
FG05554.1 	MSALRAGMAARPAVQAASRATAFRTFATSRNMRMAAVSEKTFFAG------------EPE
AN2248.1  	-----MASAFRSSLKLRASARLPAVRTITTTPRLRAA-EKPYFPN------------EPT
          	        :   ::   . .        .      :   : : ..            ** 

MG01662.1 	GPSVKTEIPGPKAQAAIKELDEVFDTRSLNMLTDYPSSSGNY~IVDPDGNVLLDV2YAQI
NCU08998.1	GPTVKTEIPGPKAKEAIKELDEVFDTRSLNMLTDYHKSVGNY~IADPDGNVLLDV2YAQI
FG05554.1 	APSVKTAIPGPKTKSHMDELTKVFDTRSANMMADYTKSKGNY~IVDPDGNVLLDV2FAQI
AN2248.1  	APKLATAIPGPKNKAASEQLNEVFDVRSLNMLADYTKSVGNY2IADLDGNMLLDV2YAQI
          	.*.: * ***** :   .:* :***.** **::** .* *** *.* ***:**** :***

MG01662.1 	ASIPIGYNNPALLEASRSPEMLQGLVNRPALGNFPPHDWTNVLKSGILKVAPKGLDQVFT
NCU08998.1	ASIPIGYNNPVLQDAARSSDMVNSIINRPALGNFPPTDWASVLKTGILKVAPKGLNQVFT
FG05554.1 	ASIAVGYNNPALLKAASSPEMVNAIVNRPALGAFPSHDWADLLKSSLLSVAPKGLDNVFT
AN2248.1  	ASIPVGYNNPHLLKVAASPEMATSLINRPALGNFPSADWAHILKTGILKVAPKGLDQVFT
          	***.:***** * ..: *.:*  .::****** **. **: :**:.:*.******::***

MG01662.1 	AMAGSDANETAYKAAFMYRRQKERGGREVPFTEEELRTSMLNKSPGASQELSIMSFKTGF
NCU08998.1	AMAGSDANETAYKAAFMWRRQKERGGPQVEFTAEEMESAMINQSPGAS-SLSILSFKSAF
FG05554.1 	AMAGSDANETAYKAAFMWRRQQERGGPDVDFSAKELDSAMNNQSPGSP-DLSILSFKTGF
AN2248.1  	AMAGSDANETAYKAAFMYYRQQQRGGPEKEFTEEEIQSSMLNQTPGSP-QLSIMSFKAGF
          	*****************: **::*** :  *: :*: ::* *::**:. .***:***:.*

MG01662.1 	HGRLFGSLSTTRSKPIHKLDIPAFDWPQATFPLLKYPLEEHAAENKAAEQAALAEVEDLI
NCU08998.1	HGRLFGSLSTTRSKPIHKLDIPAFDWPQATFPLLKYPLEEHAEENAAAEQAALDEVEHLI
FG05554.1 	HGRLFGSLSTTRSKAIHKIDIPAFDWPQATFPKLKYPLEENVEENKKIEQASIDEVEHLI
AN2248.1  	HGRLFGSLSTTRSKPIHKLDIPAFDWPQAPFPSLKYPLEEHAKENAEEEQRCLQEAERLI
          	**************.***:**********.** *******:. **   ** .: *.* **

MG01662.1 	LNYPKPPCAVIVEPIQSEGGDNHASPAFFQGLRDITKKHDVLLIVDEVQTGVGATGRFWA
NCU08998.1	KNYHIPPAAVIIEPIQSEGGDNHASPAFFRGLRAITKKHGVLLIVDEVQTGVGATGKFWA
FG05554.1 	KSWHHPPCAVVVEPVQSEGGDNHASPDFFRKLRTLTKKHNILLIVDEVQTGIGATGKFWA
AN2248.1  	KEWHNPVAAIIVEPIQSEGGDNHASPAFFRGLREITKRNNVLFIVDEVQTGVGATGKFWA
          	 .:  * .*:::**:*********** **: ** :**::.:*:********:****:***

MG01662.1 	HDHWNLSSPPDMVTFSKKAQTAGYYFGNPELRPNLPYRQFNTWMGDPARAIIFRAIIEEI
NCU08998.1	HEHWDLQDPPDMVTFSKKAQTAGYYFGNPELRPNKPYRQFNTWMGDPARALLFKAIIGEI
FG05554.1 	HEHWNLESPPDIVTFSKKAQAAGFYYADPAIRPNKPYRQFNTWMGDPARAILFKAIVDEI
AN2248.1  	HDHWNLETPPDMVTFSKKAQTAGYYFGNPALRPNKPYRQFNTWMGDPSRALIFRGIIEEI
          	*:**:*. ***:********:**:*:.:* :*** ************:**::*:.*: **

MG01662.1 	ERLDLVANTARVGDYLFANLERLAEKYPNEFQNLRGKGQGTFIAFDNPRRDEFLKRAKSF
NCU08998.1	ERLGLVEHTARVGDYLYGKLEGLAQKYPGQFANLRGKGQGTFIAFDNPRRDEFLARAKKF
FG05554.1 	NKNDLVNHTAKVGDQLFKDLEGLSKKYPGQIENLRGKGQGTFIAFDSPKRDKFLAEAKTL
AN2248.1  	ERLFLVENTAATGDYLYSGLERLAKQYPEHLQNLRGKGQGTFIAWDTPKRDEFLVKGKGV
          	::  ** :** .** *:  ** *:::** .: ************:*.*:**:** ..* .

MG01662.1 	GINIGGSGASAVRLRPMLIFQEKHA~NILLEALEKIVTSW-
NCU08998.1	GVNIGGSGQSAVRLRPMLIFEEKHA~DILVDALEKIVKSFQ
FG05554.1 	GVNIGGSGADAVRLRPMLIFQQKHA~DILLDAVEKIVKQ--
AN2248.1  	GINIGGSGQNAVRLRPMLIFQKHHA1DILLESIEKIIKQL-
          	*:****** .**********:::** :**::::***:..
```
